# TRPC6 counteracts TRPC3-Nox2 protein complex leading to attenuation of hyperglycemia-induced heart failure in mice

**DOI:** 10.1038/s41598-017-07903-4

**Published:** 2017-08-08

**Authors:** Sayaka Oda, Takuro Numaga-Tomita, Naoyuki Kitajima, Takashi Toyama, Eri Harada, Tsukasa Shimauchi, Akiyuki Nishimura, Tatsuya Ishikawa, Yoshito Kumagai, Lutz Birnbaumer, Motohiro Nishida

**Affiliations:** 1 Division of Cardiocirculatory Signaling, National Institute for Physiological Sciences (Okazaki Institute for Integrative Bioscience), National Institutes of Natural Sciences, Aichi, 444-8787 Japan; 2 Department of Physiological Sciences, SOKENDAI (School of Life Science, The Graduate University for Advanced Studies), Aichi, 444-8787 Japan; 3 Department of Translational Pharmaceutical Sciences, Graduate School of Pharmaceutical Sciences, Kyushu University, Fukuoka, 812-8582 Japan; 4 Environmental Biology Laboratory, Faculty of Medicine and Graduate School of Comprehensive Human Sciences, University of Tsukuba, Tsukuba, 305-8575 Japan; 5 Ajinomoto Co. Inc., Tokyo, 104-8315 Japan; 6 EA Pharma Co., Ltd., Tokyo, 104-0042 Japan; 7 Laboratory of Neuroscience, NIEHS, NIH, Research Triangle Park, NC 27709 USA; 8 Institute for Biomedical Research (BIOMED), Catholic University of Argentina, C1107AFF Buenos, Aires Argentina; 9 PRESTO, JST, 4-1-8 Honcho, Kawaguchi, Saitama 332-0012 Japan

## Abstract

Excess production of reactive oxygen species (ROS) caused by hyperglycemia is a major risk factor for heart failure. We previously reported that transient receptor potential canonical 3 (TRPC3) channel mediates pressure overload-induced maladaptive cardiac fibrosis by forming stably functional complex with NADPH oxidase 2 (Nox2). Although TRPC3 has been long suggested to form hetero-multimer channels with TRPC6 and function as diacylglycerol-activated cation channels coordinately, the role of TRPC6 in heart is still obscure. We here demonstrated that deletion of TRPC6 had no impact on pressure overload-induced heart failure despite inhibiting interstitial fibrosis in mice. TRPC6-deficient mouse hearts 1 week after transverse aortic constriction showed comparable increases in fibrotic gene expressions and ROS production but promoted inductions of inflammatory cytokines, compared to wild type hearts. Treatment of TRPC6-deficient mice with streptozotocin caused severe reduction of cardiac contractility with enhancing urinary and cardiac lipid peroxide levels, compared to wild type and TRPC3-deficient mice. Knockdown of TRPC6, but not TRPC3, enhanced basal expression levels of cytokines in rat cardiomyocytes. TRPC6 could interact with Nox2, but the abundance of TRPC6 was inversely correlated with that of Nox2. These results strongly suggest that Nox2 destabilization through disrupting TRPC3-Nox2 complex underlies attenuation of hyperglycemia-induced heart failure by TRPC6.

## Introduction

Heart failure is one of the major leading causes of morbidity and mortality in worldwide. Oxidative stress caused by excess accumulation of reactive oxygen species (ROS) have been suggested to mediate the development of structural and morphological changes of the heart (cardiac remodeling) induced by several risk factors including diabetic mellitus, hypertension and myocardial infarction^1^, in part through oxidative post-translational modification of intracellular signaling proteins^2^. The ROS target sulfur-containing amino acids (methionine and cysteine) on specific proteins that are found at active or allosteric sites of effector proteins^3^. In the heart, there are two major ROS-producing pathways: the mitochondrial electron transport chain and the enzymatic functions of NADPH oxidase (Nox). Mitochondria are definitely the major source of ROS production involved in the pathogenesis of heart failure^4^, but several studies have shown that inhibition of the Nox2 enzyme or Nox2 activators, such as Rac1 and p47^phox^, suppresses oxidative stress and cardiac dysfunction in mice with heart failure^5^. Upregulation of Nox2 protein has been reported to participate in cardiac fibrosis during the development of diabetic cardiomyopathy^6^. As ROS also induce mitochondrial superoxide production, so termed ‘ROS-induced ROS release (RIRR)’^7^, Nox2 may act as a primary source of ROS production and amplify RIRR signaling in heart by increasing Nox2 protein stability.

Transient receptor potential (TRP) family proteins, first described in a Drosophila visual transduction mutation *trp*, comprise 28 mammalian cation channels expressed in almost every tissue^8^. Among them, canonical TRP subfamily (TRPC) proteins, two diacylglycerol (DAG)-activated TRPC members (TRPC3 and TRPC6), have been implicated in the development of pathological cardiac remodeling^9^. TRPC3 and TRPC6 preferentially form hetero-tetramer channels^10^ and coordinately participate in angiotensin II-induced hypertrophic growth of neonatal rat cardiomyocytes (NRCMs)^11^ and pressure overload-induced cardiac hypertrophy in mice^12^. Cardiomyocyte-specific expression of TRPC3 and TRPC6 showed higher sensitivity to pressure overload-induced cardiac hypertrophy^13, 14^ and pharmacological inhibition^5, 12, 15^ or genetic deletion^12, 16^ of TRPC3 and TRPC6 attenuates heart failure in mice. We have recently reported using TRPC3-deficient mice that selective inhibition of TRPC3 is sufficient to attenuate pathological cardiac remodeling in mice^17, 18^. TRPC3 was found to positively regulate ROS signaling through increasing Nox2 protein stability by forming a protein complex with Nox2, supporting the pathological importance of TRPC3 in ROS-dependent heart failure. However, whether TRPC6 inhibition is sufficient to improve heart failure is still obscure. TRPC6 has been also reported to participate in pathological cardiac remodeling in mice with deletion of Klotho, a membrane protein predominantly produced in the kidney that exerts some antiaging effects^19^. In contrast, TRPC6 reportedly participates in physiological wound healing^16^ and negatively regulates formation of TRPC3-Nox2 complex in HEK293 cells^17^. Physiological roles of TRPC6 have been extensively studied in the kidney, but both constitutively active and dominant negative mutants of TRPC6 exacerbated renal dysfunctions^20^. This implies that TRPC6 contributes to both adaptive and maladaptive responses against environmental stress.

In this study, we demonstrate that deletion of TRPC6 failed to suppress pressure overload-induced heart failure as well as oxidative stress, despite significant attenuation of cardiac fibrosis in mice. TRPC6 deletion promotes induction of inflammatory cytokine productions in pressure-overloaded mouse hearts. In addition, hyperglycemia induced by the treatment with streptozotocin (STZ), a compound that has a preferential toxicity toward pancreatic β cells, is shown to upregulate TRPC6 in mouse heart, and that the upregulated TRPC6 negatively regulates STZ-induced oxidative stress through destabilizing Nox2 protein by counteracting the formation of the stable TRPC3-Nox2 complex.

## Results

### Deletion of TRPC6 attenuates pressure overload-induced fibrosis but not cardiac dysfunction and fibrotic gene expressions in mice

**Figure 1:**
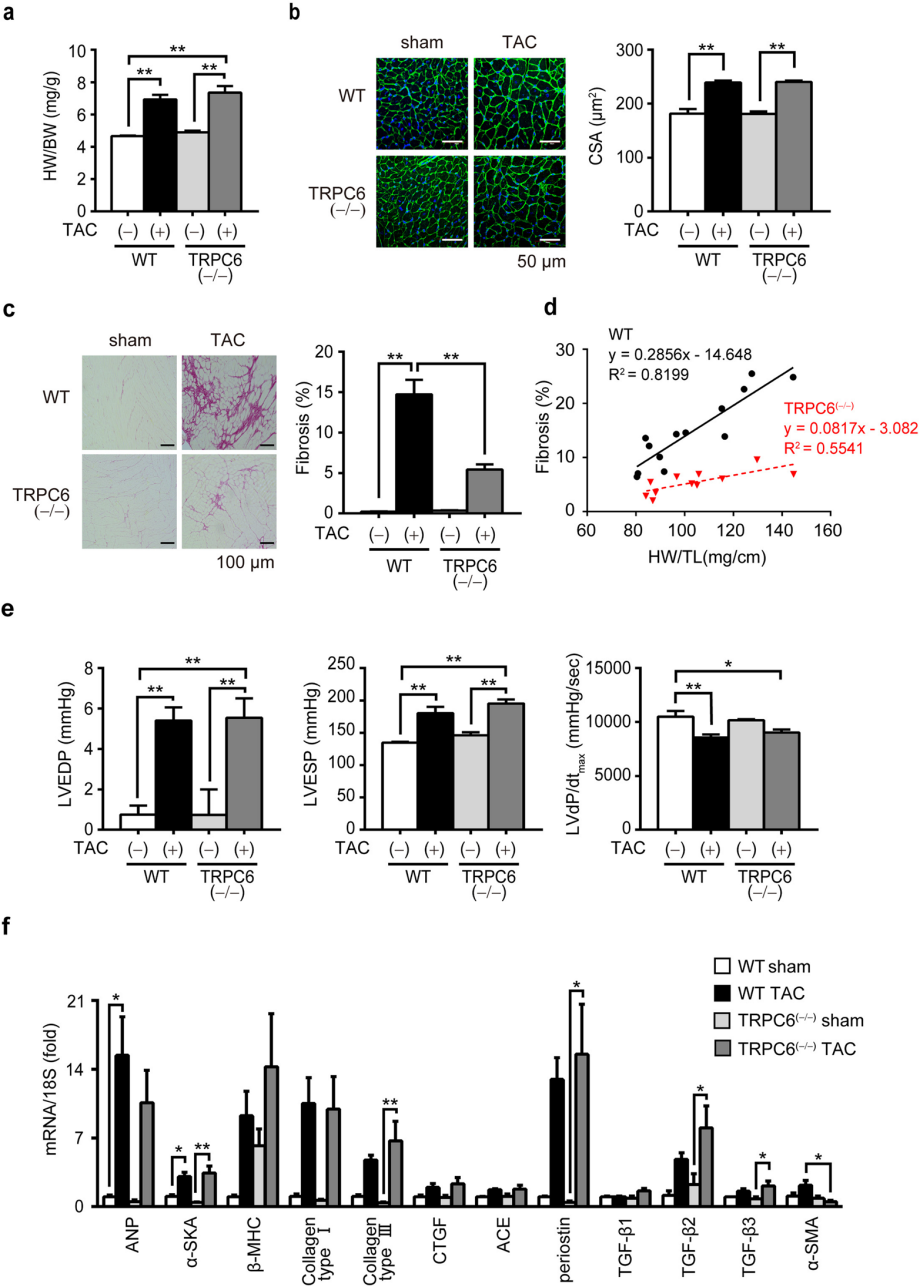
TRPC6 deletion attenuates pressure overload-induced cardiac fibrosis but not fibrotic gene expression in mice. (**a**) Heart weight (HW) /body weight (BW) ratio in WT and TRPC6^(−/−)^ mice 6 weeks after TAC. WT- TAC(−) (sham) (n = 6), TRPC6^(−/−)^-sham (n = 6), WT-TAC (n = 13), TRPC6^(−/−)^-TAC (n = 11). (**b**) Representative images of wheat germ agglutinin (WGA) staining for cross-sectional areas (CSA) measurement 6 weeks after TAC (left). Green; WGA, blue; DAPI. Quantitative results are shown in right panel. WT-sham (n = 6), TRPC6^(−/−)^-sham (n = 6), WT-TAC (n = 13), TRPC6^(−/−)^-TAC (n = 11). (**c**,**d**) TRPC6 contributes to TAC induced cardiac fibrosis. (**c**) Representative images of picrosirius red staining 6 weeks after TAC and results of interstitial fibrosis 6 weeks after TAC. WT-sham (n = 6), TRPC6^(−/−)^-sham (n = 6), WT-TAC (n = 13), TRPC6^(−/−)^-TAC (n = 11). (**d**) Relationship between fibrosis and hypertrophy 6 weeks after TAC. WT-sham (n = 6), TRPC6^(−/−)^-sham (n = 6), WT-TAC (n = 13), TRPC6^(−/−)^-TAC (n = 11). (**e**) Absence of TRPC6 does not affect pressure overload-induced LV dysfunction. LV end-diastolic pressure (LVEDP; left), LV end-systolic pressure (LVESP; middle) and LV dP/dt_max_ (right) in mice 6 weeks after TAC. WT-sham (n = 6), TRPC6^(−/−)^-sham (n = 6), WT-TAC (n = 13), TRPC6^(−/−)^-TAC (n = 11). (**f**) Expression levels of hypertrophy-related and fibrosis-related mRNAs in mouse hearts 1 week after TAC. WT-sham (n = 3), other groups (n = 4 each). Error bars, s.e.m. *P < 0.05, **P < 0.01. Results of WT mice were the same as those reported previously^17, 18^.

We first investigated whether deletion of TRPC6 also attenuates pressure overload-induced heart failure using TRPC6-deficient (TRPC6^(−/−)^) mice. Pressure overload induced by transverse aortic constriction (TAC) caused severe increases in heart weight as well as myocardial cell size in both wild type (WT) and TRPC6^(−/−)^ mice (Fig. 1a,b). TAC for 6 weeks caused severe deposition of collagen type I and type III in the interstitial area in WT hearts, and the extent of fibrosis was well correlated with that of hypertrophy (Fig. 1c,d). Surprisingly, TRPC6 deletion significantly suppressed fibrosis but not left ventricular (LV) dysfunction induced by pressure overload (Fig. 1c–e, Table 1). In contrast, increases in mRNA expression of fibrotic genes caused by 1-week TAC were never suppressed in TRPC6^(−/−)^ hearts, neither were those of hypertrophic genes (Fig. 1f). A previous report suggests that TRPC6 is highly expressed in cardiac fibroblasts and functions as a key mediator of transdifferentiation into myofibroblasts^16^. In agreement, the TAC-induced increases in mRNA expressions of α-smooth muscle actin, a reliable differentiation marker of cardiac fibroblast, were completely suppressed by TRPC6 inhibition (Fig. 1f). These results suggest that TRPC6 inhibition in cardiac fibroblasts attenuates pressure overload-induced fibrosis in mice, while TRPC6 inhibition in cardiomyocytes results in induction of cardiac dysfunction after pressure overload in mice.

**Table 1:** Cardiac parameters measured by Millar Catheter.

	WT sham (n = 6)	WT TAC (n = 13)	TRPC6 ^(−/−)^ sham (n = 6)	TRPC6 ^(−/−)^ TAC (n = 11)
Heart Rate (bpm)	410 ± 6	393 ± 6	413 ± 5	390 ± 5
LVESP (mmHg)	135 ± 1	180 ± 10 ^**^	146 ± 4	195 ± 6 ^**,†^
LVEDP (mmHg)	0.8 ± 0.5	5.4 ± 0.7 ^**^	0.7 ± 1.3	5.5 ± 1.0 ^**,†^
LVdP/dt_max_ (mmHg/sec)	10503 ± 531	8552 ± 282 ^**^	10162 ± 100	9023 ± 270 ^*^
LVdP/dt_min_ (mmHg/sec)	7298 ± 104	6598 ± 381	7397 ± 161	6269 ± 409
Tau (msec)	12.5 ± 0.3	16.0 ± 0.9	12.2 ± 0.3	19.3 ± 1.4 ^**,†^

LVESP, left ventricular end systolic pressure; LVEDP, left ventricular end diastolic pressure;

dP/dt_max_, maximal rate of pressure development; dP/dt_min_, maximal rate of decay of pressure; Tau, monoexponential time constant of relaxation.

^*^P < 0.05, ^**^P < 0.01 vs WT sham, ^†^P < 0.01 vs TRPC6 ^(−/−)^ sham. Results of WT mice were the same as those reported previously^17^.

### Inhibition of TRPC6 attenuates cardiac fibrosis but not ROS production induced by pressure overload in mice

**Figure 2:**
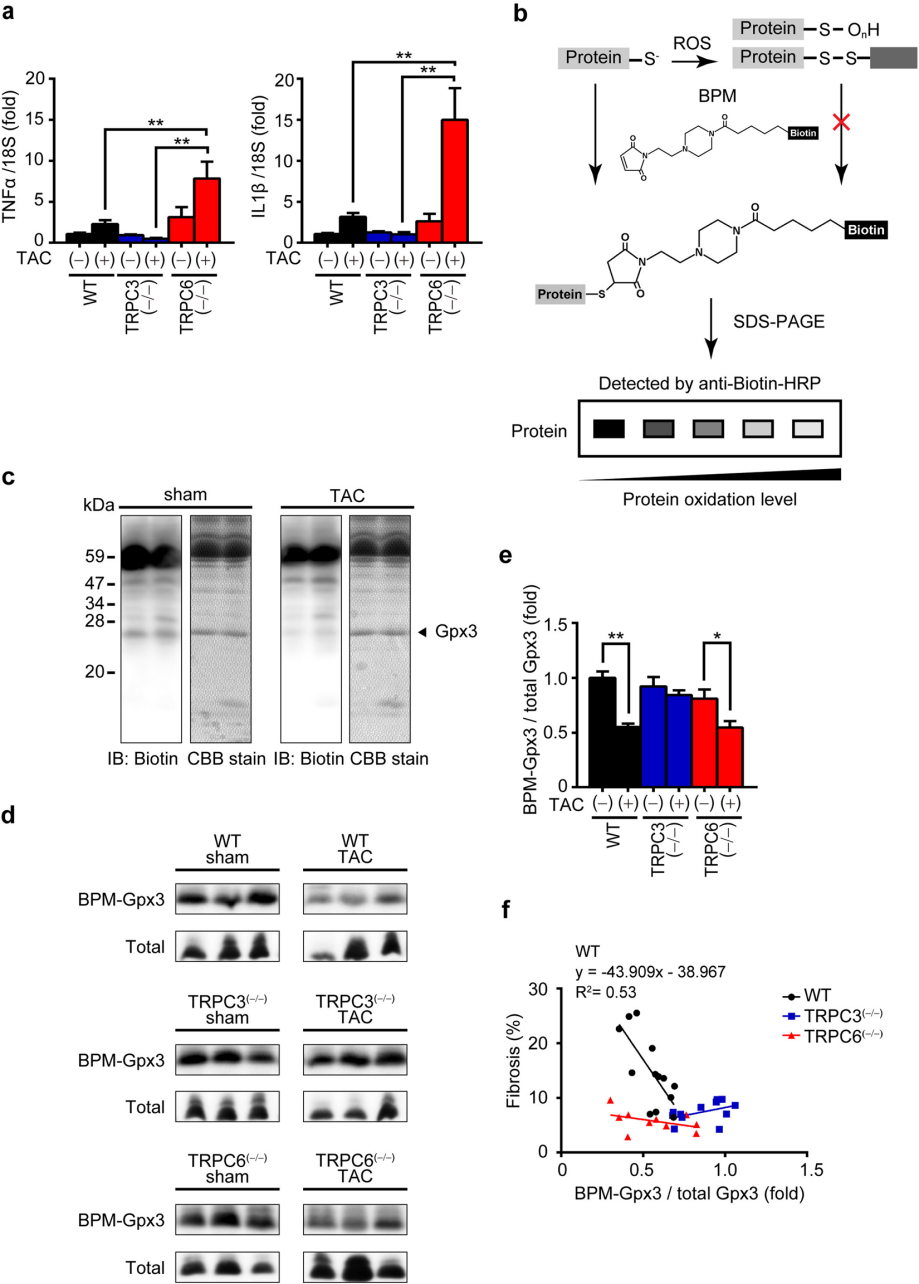
TRPC6 deletion enhances pressure overload-induced expressions of inflammatory cytokine mRNAs in mouse hearts. (**a**) Expression levels of TNFα and IL1β mRNAs in mouse hearts 1 week after TAC. WT sham (n = 3), other groups (n = 4 each). (**b**) Schema for the principle of BPM labeling assay. (**c**) Representative images of western blotting using anti-biotin-HRP and coomassie brilliant blue (CBB) staining of gels loaded with plasma samples from mice 6 weeks after TAC. Full-length blots and gels are presented in Supplementary Fig. 2a. (**d**,**e**) Representative western blot (**d**) and quantification (**e**) of BPM-modified and total Gpx3 in mouse plasma 6 weeks after TAC. WT-sham (n = 6), TRPC3^(−/−)^-sham (n = 6), TRPC6^(−/−)^-sham (n = 6), WT-TAC (n = 13), TRPC6^(−/−)^-TAC (n = 12), TRPC6^(−/−)^-TAC (n = 11). Full-length blots of all samples are presented in Supplementary Fig. 2b. (**f**) Relationship between fibrosis and levels of BPM-modified Gpx3. WT-sham (n = 6), TRPC3^(−/−)^-sham (n = 6), TRPC6^(−/−)^-sham (n = 6), WT-TAC (n = 13), TRPC3^(−/−)^-TAC (n = 12), TRPC6^(−/−)^-TAC (n = 11). Error bars, s.e.m. ^*^P < 0.05, ^**^P < 0.01.

We next investigated whether inhibition of TRPC6 in cardiomyocytes enhances inflammatory response in pressure-overloaded mouse hearts. The mRNA expression levels of interleukin (IL)1β and tumor necrosis factor (TNF)α in TAC-operated TRPC6^(−/−)^ hearts were significantly higher than those in WT and TRPC3^(−/−)^ hearts (Fig. 2a), indicating enhancement of inflammatory cytokine production by TRPC6 inhibition. Oxidative stress due to increased Nox2 activity has been implicated in sepsis-induced cardiac inflammation^21^, and TRPC3 positively regulates ROS signaling through stabilizing and activating Nox2 in rodent heart^17^. However, we previously confirmed that Nox2 expression levels were not enhanced in pressure-overloaded TRPC6^(−/−)^ hearts and TRPC6 apparently has no impact on Nox2 stability and activity in rodent cardiomyocytes. Therefore, we examined whether inhibition of TRPC6 promotes ROS production from the heart. In order to reduce the number of animal experiment, we developed a new method to evaluate cardiac ROS production indirectly by measuring ROS-dependent oxidative modification of cysteine thiol on plasma proteins in arterial blood. Using biotin-PEAC_5_-maleimide (BPM) as a competitive electrophile to react with free cysteine thiol (Fig. 2b), we found that the intensity of a single band below 30 kDa observed in sham-operated heart was dramatically reduced in TAC-operated heart (Fig. 2c). Mass spectroscopic analysis with Mascot software revealed that this protein was identified as glutathione peroxidase 3 (Gpx3), a plasma-specific enzyme that reduces lipid hydroperoxides and hydrogen peroxide^22^ (Supplementary Fig. 1). Using BPM-dependent modification of Gpx3 (BPM-Gpx3) as an indirect marker of reducing status in blood, we found that TAC significantly reduced BPM-Gpx3 band intensity in the plasma from WT mice, which was well correlated with the severity of fibrosis (Fig. 2d–f). BPM-Gpx3 intensities were higher in the plasma from TAC-operated TRPC3^(−/−)^ mice, suggesting the reduction of oxidative stress in the artery. However, BPM-Gpx3 intensities were also reduced in the plasma from TRPC6^(−/−)^ mice to the same extent as that from WT mice. These results suggest that suppression of pressure overload-induced cardiac fibrosis by TRPC6 inhibition is absolutely independent of ROS production, and that suppression of fibrosis compensatively promotes pressure overload-induced inflammatory cytokine expressions in mouse hearts.

### Inhibition of TRPC6 exacerbates STZ-induced cardiac dysfunction

**Figure 3:**
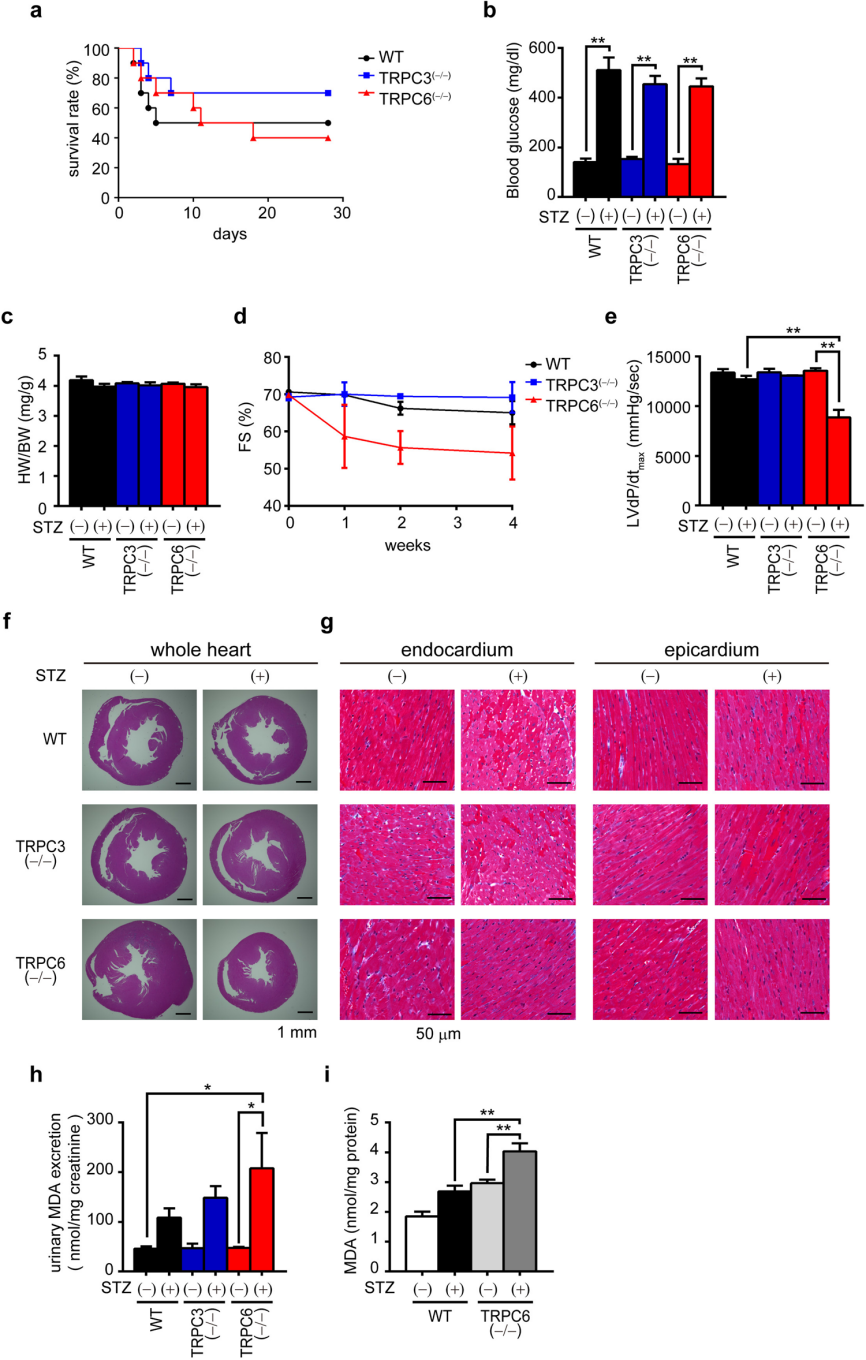
Deletion of TRPC6 but not TRPC3 promotes streptozotocin(STZ)-induced cardiac dysfunction in mice. (**a**) Survival rate of STZ-treated mice (n = 10 each). (**b**) Concentration of blood glucose (n = 3 each). (**c**) HW/BW ratio of mice with or without STZ treatment for 4 weeks (n = 3 each). (**d**,**e**) Absence of TRPC6 exacerbates STZ-induced LV dysfunction. Time courses of fractional shortening (FS) (**d**) and LVdP/dt_max_ at the end of 4-week STZ treatment (**e**) (n = 3 each). (**f**,**g**) Representative images of hematoxylin & eosin-stained LV sections to visualize cross sectional area of cardiomyocytes (**f**) and magnified endocardial (left) and epicardial (right) regions of LV sections stained with Masson Trichrome (**g**). (**h**,**i**) Absence of TRPC6 enhances hyperglycemia-induced ROS production. Accumulation of malondialdehyde (MDA) in urine (**h**) and heart (**i**) (n = 3 each). Error bars, s.e.m. ^*^P < 0.05,^**^P < 0.01.

Diabetes mellitus is one of the most important risk factors for heart failure and major cause of increased morbidity and mortality^23^. Inflammatory mechanisms, including oxidative stress and cytokine productions have been proposed to participate in the cardiovascular diabetic complication^23–25^. Treatment with STZ increased mortality rate in both WT and TRPC6^(−/−)^ mice compared to TRPC3^(−/−)^ mice (Fig. 3a), although all mice showed significant increases in blood glucose levels at the same extent (Fig. 3b). The STZ treatment had no impact on heart weight in WT, TRPC3^(−/−)^ and TRPC6^(−/−)^ mice (Fig. 3c), while LV contractility was significantly reduced in STZ-treated TRPC6^(−/−)^ mice (Fig. 3d,e). Treatment of all mice with STZ never caused apparent structural remodeling such as hypertrophy and fibrosis (Fig. 3f,g), but it significantly increased urinary malondialdehyde (MDA) concentration in TRPC6^(−/−)^ mice compared to those in WT and TRPC3^(−/−)^ mice (Fig. 3h). Accordingly, TRPC6 deletion significantly increased plasma levels of total cholesterol (TCHO) and high density lipoprotein cholesterol (HDLC), and urinary protein, aldosterone and corticosterone levels induced by STZ treatment (Table 2). Cardio-renal relationships have now attracted attention as an important mechanism underlying maintenance and transfiguration of cardiac homeostasis. These results strongly suggest that TRPC6 deletion exacerbates not only hyperglycemia-induced cardiac dysfunction but also renal dysfunction in mice. Treatment with STZ at lower dose (50 mg/kg) failed to cause cardiac dysfunction in TRPC6^(−/−)^ mice despite significant increase in blood glucose level equivalent to that in high-dose STZ-treated mice (>400 mg/dl), but significantly potentiated hyperglycemia-induced increase in cardiac MDA concentration in WT hearts (Fig. 3i). These results indicate that inhibition of TRPC6 exacerbates diabetic heart failure induced by STZ in mice.

**Table 2:** Results of plasma and urinary parameters in STZ-treated mice.

		WT STZ(−) (n = 3)	WT STZ( + ) (n = 3)	TRPC3^(−/−)^ STZ(−) (n = 3)	TRPC3^(−/−)^ STZ( + ) (n = 3)	TRPC6^(−/−)^ STZ(−) (n = 3)	TRPC6^(−/−)^ STZ( + ) (n = 3)
plasma	GLU (mg/dl)	140 ± 14	511 ± 51^**^	153 ± 9	454 ± 34^**^	133 ± 21	445 ± 32^**^
	TCHO (mg/dl)	69 ± 6	146 ± 26	59 ± 6	131 ± 14	62 ± 10	203 ± 62^†^
	HDLC (mg/dl)	61 ± 5	134 ± 21	59 ± 4	105 ± 2	51 ± 7	166 ± 48^*,†^
urine	Protein (mg/mg Cre)	70.4 ± 10.6	111.5 ± 15.1	46.2 ± 1.2	128.5 ± 14.9	38.9 ± 4.6	267.7 ± 92.4^*^
	Aldosterone (ng/mg Cre)	8.2 ± 0.22	62.0 ± 17.3	16.5 ± 5.0	41.3 ± 14.1	39.3 ± 19.2	253.8 ± 109.9^*^
	Corticosterone (ng/mg Cre)	56.1 ± 3.4	711.8 ± 261.9	122.3 ± 57.0	259.8 ± 69.1	184.0 ± 39.8	2431.3 ± 941.4^*^

GLU, glucose; TCHO, total cholesterol; HDLC, high density lipoprotein cholesterol; Cre, creatinine﻿.

^*^P < 0.05, ^**^P < 0.01 vs WT STZ(−), and ^†^P < 0.05 vs TRPC6 ^(−/−)^ STZ(−).

### Hyperglycemia-induced TRPC6 upregulation reduces risk for diabetic heart failure by counteracting TRPC3-Nox2 protein complex

**Figure 4:**
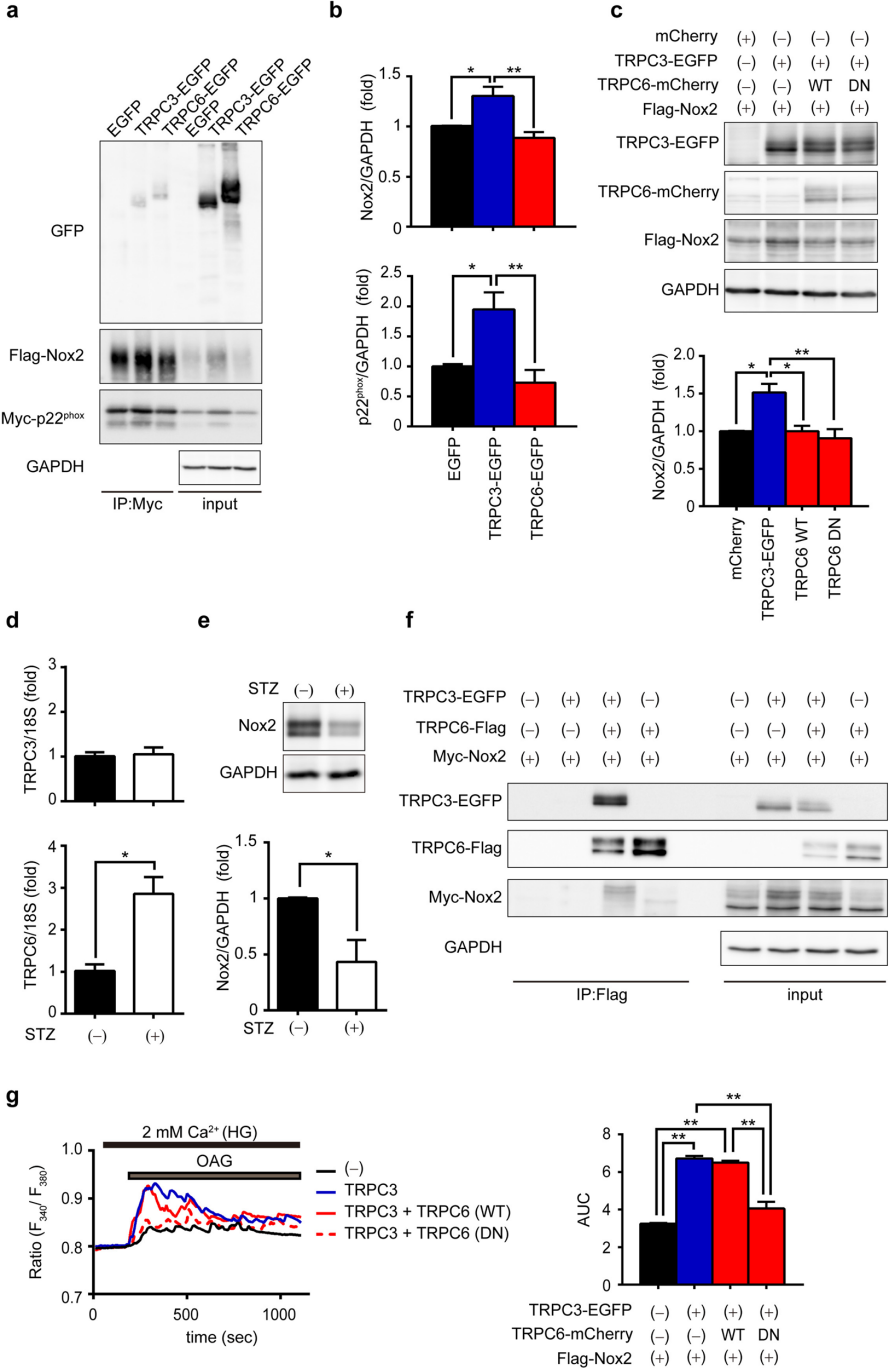
Involvement of TRPC6 in hyperglycemia-induced Nox2 downregulation through inhibition of the TRPC3-Nox2 complex formation. (**a**) Interaction of TRPC6-EGFP with Flag-Nox2 and Myc-p22^phox^ in HEK293 cells. Immunoprecipitation (IP) was performed using an anti-Myc antibody. Full-length blots are presented in Supplementary Fig. 3a. (**b**) Quantitative results of Nox2 and p22^phox^ protein abundances in input samples. (n = 4 each) (**c**) Effects of expression of TRPC6 (WT or DN) on Nox2 stability in TRPC3-EGFP and Flag-Nox2-expressing HEK293 cells (n = 3 each). Full-length blots are presented in Supplementary Fig. 3b. (**d**) Expression levels of TRPC3 and TRPC6 mRNAs in mouse hearts with or without STZ treatment (n = 3 each). (**e**) Nox2 protein abundance in mouse hearts with or without STZ (n = 3 each). Full-length blots are presented in Supplementary Fig. 3c. (**f**) Formation of protein complex among TRPC3-EGFP, Myc-Nox2 and TRPC6-Flag. IP was performed using anti-Flag antibody. Full-length blots are presented in Supplementary Fig. 3d. (**g**) OAG (90 µM)-induced Ca^2+^ responses in HEK293 cells expressing EGFP (−), TRPC3-EGFP (TRPC3), TRPC3-EGFP and TRPC6-WT-mCherry (TRPC3 + TRPC6(WT)), and TRPC3-EGFP and TRPC6-DN-mCherry (TRPC3 + TRPC6(DN)). Averaged time courses (left) and [Ca^2+^]_i_ increases shown as area under the curve (AUC, right) (n = 30 cells). Error bars, s.e.m. ^*^P < 0.05, ^**^P < 0.01.

We next examined whether TRPC6 negatively regulates Nox2 stability through disrupting TRPC3-Nox2 complex in diabetic mouse hearts. Immunoprecipitation analysis using myc-tagged p22^phox^ (Myc-p22^phox^), an essential partner of Nox2 to form stable heteromeric complex, revealed that Nox2 protein could interact with TRPC6 as well as TRPC3 (Fig. 4a). However, we previously reported that co-expression of TRPC6 with TRPC3 and Nox2 canceled TRPC3-dependent Nox2 stabilization in HEK293 cells^17^. We also confirmed that overexpression of TRPC6 alone had no impact on basal Nox2 stability in flag-tagged Nox2 (Flag-Nox2)-expressing HEK293 cells, excluding the possibility that TRPC6 itself directly destabilize Nox2 protein (Fig. 4a,b). In addition, the Nox2 upregulation via formation of TRPC3-EGFP / Flag-Nox2 protein complex was significantly suppressed by overexpression of pore-dead (with deleted LFW motif)^10^ dominant negative mutant of TRPC6 (TRPC6-DN), as well as wild type TRPC6 (TRPC6-WT) (Fig. 4c). These results strongly suggest that TRPC6 counteracts the TRPC3-Nox2 protein complex to destabilize Nox2 protein. Hyperglycemia induced by STZ significantly increased mRNA expression level of TRPC6, but not that of TRPC3, in mouse heart (Fig. 4d). The abundance of Nox2 protein was decreased by STZ treatment in WT hearts (Fig. 4e). Furthermore, immunoprecipitation analysis revealed that destabilization of the Nox2 in TRPC3-EGFP and Myc-Nox2-expressing HEK293 cells was correlated with the enhanced expression of TRPC6 as well as the interaction of TRPC6 with TRPC3 (Fig. 4f). We also confirmed that HEK293 cells co-expressing TRPC3-EGFP and TRPC6-DN mutant never showed Ca^2+^ responses evoked by 1-oleoyl-2-acetyl-*sn*-glycerol (OAG), a DAG-derivative that can directly increase TRPC6 channel activity (Fig. 4g). These results suggest that TRPC6 negatively regulates hyperglycemia-induced Nox2 stabilization through counteracting and disrupting formation of TRPC3-Nox2 stable protein complex in heart.

### TRPC6 negatively regulates high glucose-dependent basal cytokine production independently of Ca^2+^ channel activity in NRCMs

**Figure 5:**
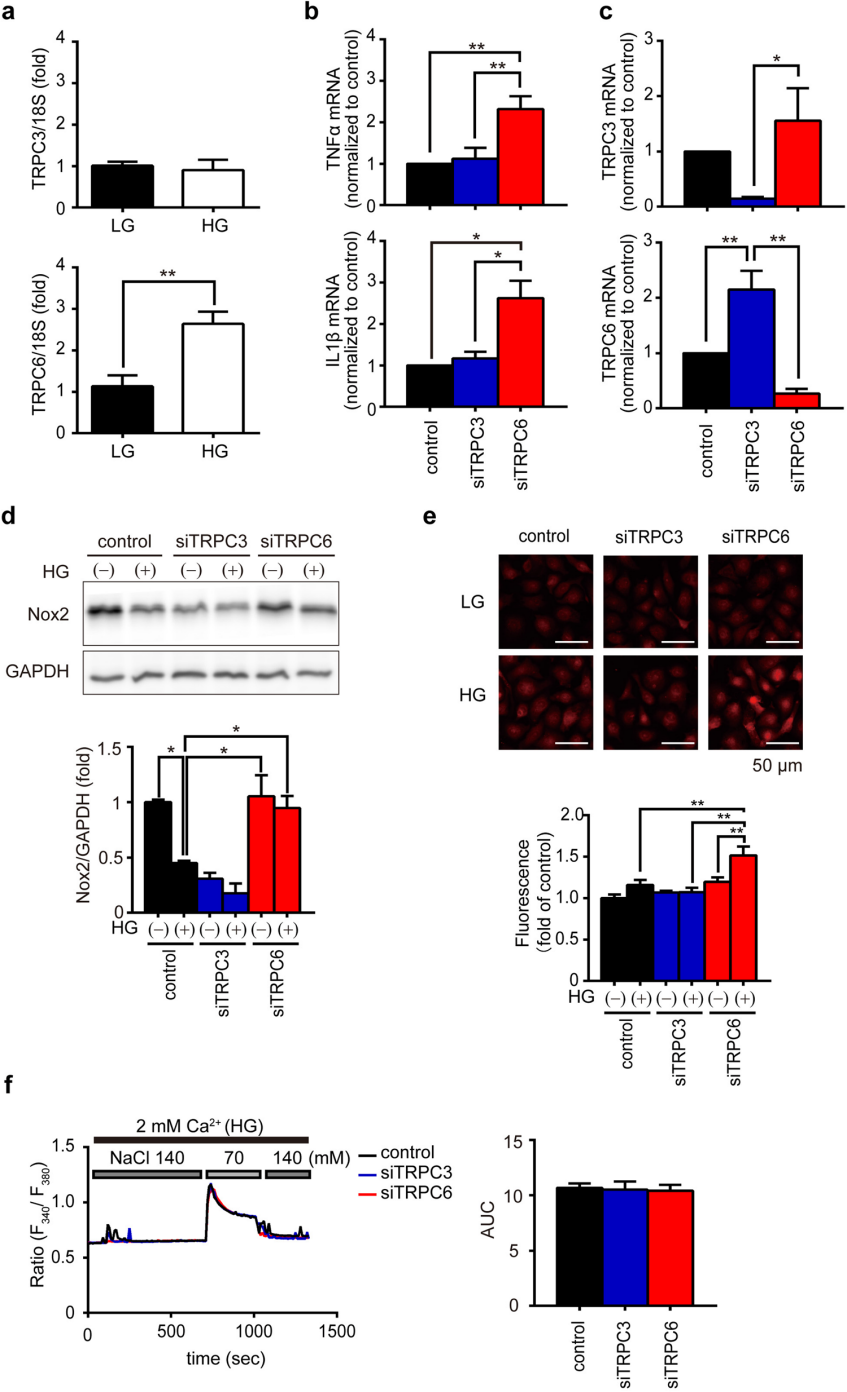
Involvement of TRPC6 in TRPC3/Nox2-mediated ROS production. (**a**) Expression levels of TRPC3 and TRPC6 mRNAs in NRCMs cultured in low (LG, 5.5 mM) or high (HG, 25 mM) glucose-containing medium (n = 3 each). (**b**) Expression levels of TNFα or IL1β in NRCMs cultured in LG or HG medium (n = 7 each). (**c**) Expression levels of TRPC3 and TRPC6 mRNAs in NRCMs transfected with siRNAs. (n = 5 each). (**d**,**e**) Abundance of Nox2 protein (**d**) and ROS production (**e**) in siRNA-transfected NRCMs cultured in LG or HG medium (n = 3 each). Full-length blots are presented in Supplementary Fig. 4. (**f**) Ca^2+^ responses induced by hypotonic stress (70 mM NaCl) in siRNA-transfected NRCMs. Averaged time courses (left) and [Ca^2+^]_i_ increases shown as AUC (right) (n = 45–46 cells). Error bars, s.e.m.^*^P < 0.05, ^**^P < 0.01.

We further examined whether TRPC6 negatively regulates hyperglycemia-induced inflammatory cytokine production in NRCMs. As NRCMs are normally cultured in high-glucose (25 mM) medium, we first investigated whether TRPC6 expression level is upregulated in NRCMs with high-glucose medium compared to those with low-glucose (5.5 mM) medium, because NRCMs in low-glucose medium are vulnerable to hypoxia-induced ROS-mediated mitochondrial injury^26^. As expected, TRPC6 mRNA expression level is higher in NRCMs with high glucose medium than those with low-glucose medium (Fig. 5a). In addition, knockdown of TRPC6, but not TRPC3, significantly increased basal mRNA expression levels of IL1β and TNFα in NRCMs with high-glucose medium (Fig. 5b). Knockdown of TRPC6 compensatively increased TRPC3 mRNA expression level, as well as ROS production and Nox2 protein abundance in NRCMs when cultured in the presence of high glucose (Fig. 5c–e). These results strongly suggest that high-glucose-dependent TRPC6 upregulation negatively regulates basal inflammatory cytokine production through inhibiting TRPC3-Nox2-mediated ROS production in NRCMs.

TRPC6 channel activity is reportedly enhanced by mechanical stress^27, 28^. Mechanical stretching of NRCMs induced by replacement of normal high-glucose solution to hypotonic solution increased intracellular Ca^2+^ concentration ([Ca^2+^]_i_), while this [Ca^2+^]_i_ increase was not suppressed by knockdown of TRPC3 or TRPC6 (Fig. 5f). As the basal [Ca^2+^]_i_ in TRPC6-silenced NRCMs were similar with that in control NRCMs, this result indicates that high glucose upregulates TRPC6 level but never increases TRPC6 channel activity in NRCMs, and suggests that TRPC6 upregulation, but not TRPC6 channel activity, underlies high-glucose-dependent negative regulation of basal inflammatory cytokine production in NRCMs.

## Discussion

**Figure 6:**
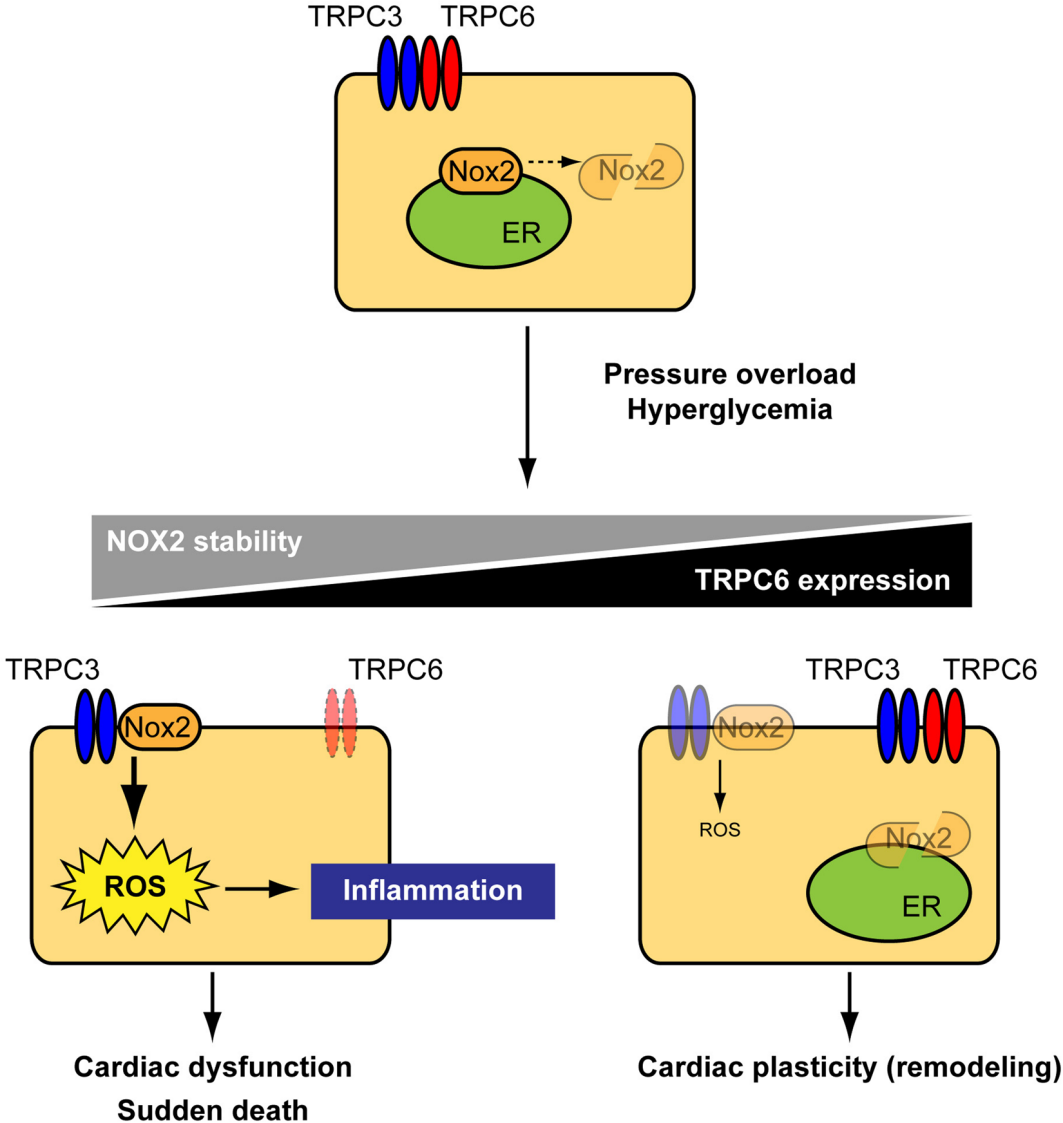
Schema of negative crosstalk between TRPC6 and TRPC3-Nox2 complex in cardiomyocytes. In resting condition, TRPC3 and TRPC6 channels function independently or coordinately in cardiomyocytes. Once hearts are exposed to environmental stresses such as hemodynamic load and hyperglycemia, TRPC3 forms stable protein complex with Nox2, which evokes aberrant ROS production in cardiomyocytes. In contrast, environmental stresses also upregulate TRPC6, which can counteract formation of the TRPC3-Nox2 complex in cardiomyocytes, leading to Nox2 destabilization, and resulting in negative regulation of ROS signaling in heart.

The TRPC family includes seven members, and is divided into two groups based on structural and functional similarities: TRPC1/4/5, which are sensitive to inositol-1,4,5-trisphosphate (IP_3_)-mediated Ca^2+^ release from IP_3_-sensitive Ca^2+^ store followed by capacitative Ca^2+^ entry triggered by Ca^2+^ depletion in the intracellular Ca^2+^ store, and TRPC3/6/7, which are sensitive to DAG. TRPC4^29^ and TRPC5^30^ are activated by intracellular Ca^2+^, TRPC1 is activated by store depletion^30^, and TRPC4 and TRPC5 are also activated by DAG in an Na^+^/H^+^ exchanger regulatory factor-dependent manner^31^. TRPC6 protein preferentially associates with TRPC3 and TRPC7 proteins to form DAG-activated homo- and/or hetero-multimer channels^32^. Although TRPC3 (as well as TRPC7) and TRPC6 are 75% identical and higher degree of functional similarity, TRPC3 and TRPC6 differ substantially in their basal channel activities. TRPC6 is a tightly regulated receptor-activated cation channel^33^, while TRPC3 and TRPC7 display considerable constitutive activity^34^. A discrepant N-linked glycosylation pattern has been reported to determine the differences between basal TRPC3 and TRPC6 channel activities^33^. TRPC3, but not TRPC6, acts as a signaling platform through interacting with phospholipase C^35^ and protein kinase C^36^ to amplify agonist-induced intracellular signaling pathways. TRPC6^(−/−)^ mice were originally shown an increased vascular smooth muscle contractility through compensative upregulation of TRPC3^37^, suggesting that TRPC6 has distinct nonredundant roles in the control of vascular smooth muscle tone. Using TRPC6-deficient 129/﻿Sv background mice, we found that TRPC6 deletion failed to suppress pressure overload-induced LV dysfunction as well as oxidative modification of plasma Gpx3 protein, despite significant suppression of interstitial fibrosis (Figs. 1 and 2). This result suggests that inhibition of cardiac fibroblast differentiation into myoblast is insufficient to suppress heart failure. In addition, deletion of TRPC6 promoted STZ-induced sudden death as well as cardiac dysfunction and oxidative stress (Fig. 3). High glucose upregulates TRPC6 and destabilize Nox2 protein in NRCMs, and knockdown of TRPC6, but not TRPC3, enhanced basal ROS production as well as inflammatory cytokine production, independently of TRPC6 channel activity (Fig. 5). These results strongly suggest that TRPC6 negatively regulates ROS signaling through counteracting the TRPC3-Nox2 complex in rodent cardiomyocytes (Fig. 6).

The mRNA expression levels of IL1β and TNFα were upregulated in TAC-operated TRPC6^(−/−)^ hearts compared to those in WT and TRPC3^(−/−)^ hearts (Fig. 2). It is well known that inductions of these genes depend on nuclear factor (NF)-κB activity. We have previously reported that purinergic P2Y_6_ receptor-α subunit of G_12/13_ (Gα_12/13_) protein signaling initiates TAC-induced cardiac fibrosis in mice^38^, and the activation of Gα_13_ increases expression of these cytokines in a NF-κB-dependent manner in rat cardiac fibroblasts^39^. The Gα_13_ activation also increases TRPC6 expression levels, while the upregulated TRPC6 proteins negatively regulate fibrotic responses of cardiac fibroblasts^40^. These findings suggest that Gα_12/13_-mediated NF-κB-dependent signaling pathway may be potentiated in TAC-operated TRPC6^(−/−)^ hearts.

Focusing on the redox status in the blood of mice with heart failure, we demonstrated that oxidative modification of plasma Gpx3 protein was well correlated with the severity of maladaptive cardiac remodeling induced by pressure overload (Fig. 2). Because TRPC6 deletion suppressed cardiac fibrosis without suppressing Gpx3 oxidation, the extent of Gpx3 oxidative modification seems not necessarily correlated with the severity of interstitial fibrosis in mice. However, oxidative modification of Gpx3 could be a good marker for estimating ROS production in heart through the TRPC3-Nox2 complex, since TRPC3-deficient mice showed a complete reduction of Gpx3 oxidation as well as fibrosis. As the inhibition of TRPC3, but not TRPC6, actually suppressed pressure overload-induced heart failure, oxidative modification of Gpx3 will be a novel biomarker for diagnosing the severity of maladaptive cardiac remodeling.

Gpx3 contains a selenocysteine (Sec) residue at its active center site, and Sec is highly nucleophilic and easily react with electrophilic molecules, including peroxide. As the deprivation of this Sec results in reduced Gpx3 enzymatic activity, electrophilic modification of this Sec will also reduce Gpx3 enzymatic activity. However, we could not observe any apparent reduction of plasma peroxidase activity in mice (data not shown). This result suggests that BPM does not react with Sec, but may target one of other two cysteine thiols in Gpx3. Further study will be necessary to reveal the physiological significance of electrophilic modification of Gpx3 during the development of heart failure.

In conclusion, we revealed a new channel activity-independent role of TRPC6 protein in murine cardiac pathology. Upregulation of TRPC6 protein destabilizes the TRPC3-Nox2 complex, which leads to prevention of ROS production-dependent cardiac dysfunction induced by hyperglycemia. Elucidation of the role of TRPC6 as an endogenous negative regulator of ROS signaling deepens the understanding of the molecular diversity and function of TRPC channels and will provide a novel therapeutic strategy for heart failure.

## Methods

### Animals and reagents

All protocols using mice and rats were reviewed and approved by the ethics committees at the National Institutes of Natural Sciences or the Animal Care and Use Committee, Kyushu University, and were performed according to the institutional guidelines concerning the care and handling of experimental animals. 129/﻿Sv mice with homozygous deletion of the gene encoding TRPC3 and TRPC6 were provided by the Comparative Medicine Branch, National Institute of Environmental Health Sciences, Research Triangle Park, North Carolina 27709. Genotyping was performed as previously described^32^. Mice were maintained in specific-pathogen-free area under a 12-hour/12-hour light/dark cycle. Sprague-Dawley rats were purchased from Japan SLC, Inc. STZ and lipopolysaccharide (LPS) were purchased from Sigma. Alexa Fluor 488-conjugated wheat germ agglutinin (WGA) was purchased from Life technologies.

### TAC surgery and STZ treatment, and measurement of cardiac functions

Pressure overload was induced as described previously^5, 17^. Male mice, 6–8 weeks old, were used for these experiments. Cardiac pressure overload was induced by TAC. Briefly, mice were anesthetized using a mixture of domitor (Zenoaq), midazolam (Sando) and butorphanol (Meiji Seika Pharma). After orotracheal intubation and ventilation, an intercostal space was opened. The transverse aorta was then exposed and constricted between the brachiocephalic artery and left carotid artery to the width of a 27-G needle using a 5–0 silk braid. Sham treatment was performed similarly but without constriction of the silk braid.

For the development of diabetic heart failure, male 129﻿/﻿Sv mice (10–12-week-old) were injected intraperitoneally with STZ (250 mg/kg, dissolved in sodium citrate buffer (0.1 M, pH 4.5)). For cardiac MDA measurement, male 129/Sv mice were injected with minimal STZ (50 mg/kg) for 5 successive days, and then, LPS (5 mg/kg) dissolved in phosphate buffered saline were injected intraperitoneally in mice 4 weeks after STZ treatment to promote hyperglycemia-dependent inflammation. Mice were sacrificed 24 hours after LPS injection.

### Measurement of LV functions and biochemical parameters

Echocardiography and catheterization were performed as described previously^5, 17^. Echocardiography was performed using Nemio-XG echocardiography (Toshiba) with 14-MHz transducer under anesthesia with isoflurane (induction: 3–4%). LV hemodynamic parameters of mice 6-week after TAC or 4-week STZ treatment were assessed using a micronanometer catheter (Millar 1.4F, SPR 671, Millar Instruments) under anesthesia. Cardiac parameters of WT animals have been already reported^17^.

Measurement of biochemical parameters in plasma glucose (Glucose Pilot, Technicon), total cholesterol, and high density lipoprotein cholesterol, and urinary protein (Pierce BCA protein assay kit, Thermo Fisher Scientific), urinary aldosterone and corticosterone (Enzyme immunoassay system, Cayman Chemical) were performed as described previously^41^. Blood samples collected from cardiac apex at the time of euthanasia were centrifuged (3,000 × g) for 15 min to prepare plasma samples. Plasma total cholesterol (TCHO) and HDL cholesterol (HDLC) levels were measured using DRI-CHEM 7000Z (Fuji Film) with slides of TCHO-PIII or HDL-C-PIIID (Fuji Film) according to manufacturer’s instruction. Mice were individually placed in metabolic cages (Natsume), and urine samples were collected for 24 hours.

### Morphological analysis of mouse hearts

Staining of LV sections (3 μm thickness) with hematoxylin and eosin, Masson trichrome and picrosirius red were described previously^17, 18, 38^. To assess CSA of cardiomyocytes, the sections were stained with Alexa Fluor 488-conjugated WGA and DAPI (Dojindo). Three regions were selected at random for each left ventricle, and the average values were calculated using a BZ-II Analyzer (Keyence).

### Cell cultures and isolation of NRCMs

HEK293 cells were cultured in DMEM supplemented with 10% FBS and 1% penicillin and streptomycin. Plasmid DNAs were transfected into HEK293 cells with X-tremeGENE9 (Roche) according to manufacturer’s instruction. Isolation of NRCMs were performed as described previously^17^. After serum deprivation, NRCMs were cultured in either normal high-glucose DMEM (25 mM) or low glucose DMEM (5.5 mM). For protein knockdown, cells were transfected with siRNAs^11, 17^ (10 nM) using Lipofectamine RNAiMAX (Invitrogen) for 72 hours.

### Measuring mRNA expression in cells and tissues

Total RNA was isolated from frozen mouse heart samples using an RNeasy Fibrous Tissue Mini Kit (Qiagen) or from cardiomyocytes using an RNeasy Mini Kit (Qiagen) as described previously^17^. Quantitative real-time PCR (qRT-PCR) was performed using Lightcycler^®^ 96 (Roche) with OneStep RT-PCR Kit (Qiagen) or KAPA SYBR^®^ FAST qPCR kit (KAPA BIOSYSTEMS). All Taqman probes were purchased from Applied Biosystems^18^. Primers used in qRT-PCR with SYBR Green were as follows: mouse IL1β forward 5′-CACCTTCTTTTCCTTCATCTTTG-3′, reverse 5′-GTCGTTGCTTGTCTCTCCTTGTA-3′; mouse TNFα forward 5′-ACTGAACTTCGGGGTGATTG-3′, reverse 5′-GCTTGGTGGTTTGCTACGAC-3′; mouse TRPC6 forward 5′-GACCGTTCATGAAGTTTGTAGCAC-3′, reverse 5′-AGTATTCTTTGGGGCCTTGAGTCC-3′; rodents 18 S ribosomal RNA forward 5′-ATTAATCAAGAACGAAAGTCGGAGGT-3′, reverse 5′-TTTAAGTTTCAGCTTTGCAACCACACT-3′.

### Measurement of intracellular Ca^2+^ increases and ROS production

Measurement of intracellular Ca^2+^ increases were performed with Fura 2-AM (Dojindo) as previously described^42^. After aspirating the culture medium from the dishes and washing the cells with DMEM, freshly prepared 1 μM Fura 2-AM diluted in DMEM was added to the dishes and incubated for 30 min at room temperature. The dye solution was then replaced with HEPES-buffered saline solution (HBSS) containing 140 mM NaCl, 5.6 mM KCl, 5.5 mM or 25 mM glucose, 10 mM HEPES (pH 7.4), 1 mM MgCl_2_ and 2 mM CaCl_2_. Fluorescence images were recorded and analyzed using a video image analysis system (Metafluor, Molecular Devices). Only NaCl concentration was reduced to 70 mM in hypotonic buffer. Dihydroethidium (DHE) staining with NRCMs was performed as previously described^5^.

### Measurement of MDA concentration

Total cardiac and urinary MDA was assessed in mice using a Lipid peroxidation (MDA) Assay kit (Abcam) as described previously^17^. Briefly, frozen mouse heart samples were weighed and homogenized in MDA Lysis Buffer and buthylated hydroxytoluene, and the lysates were clarified by centrifugation at 13,000 × g for 10 min at 4 °C. Samples of cardiac supernatant and urine samples were then allowed to react with thiobarbituric acid reagent at an acidic pH at 95 °C for 1 hour and read at 532 nm using a Spectra Max i3 (Molecular Devices). MDA concentrations were estimated using a standard curve derived using standard MDA. Total cardiac protein concentration was determined by DC^TM^ protein assay (BIO-RAD).

### Western blotting

To analyze expression of endogenous Nox2 in mouse hearts and NRCMs, total membrane fraction was isolated as described previously^36^. Mouse hearts were first powdered in liquid nitrogen and then lysed in hypotonic lysis buffer. Co-immunoprecipitation of TRPC6 with Flag-Nox2 and Myc-p22^phox^ was performed as previously described^17^. Primary antibodies used were; GAPDH (sc-25778), gp91^phox^ (sc-130543), from Santa Cruz Biotechnology, Flag M2-HRP (A8592) from Sigma Aldrich, GFP (CHIP grade, ab290) from Abcam, Myc-tag (05–742) from Merck and TRPC6 from cell signaling technologies.

### Identification of BPM-bound plasma proteins

Competitive BPM labeling assay was performed as described previously^43^. Briefly, mouse plasma (20 μg of protein) were prepared and mixed with biotin-PEAC_5_-maleimide (BPM, Dojindo) (25 µM) and rocked at 37 °C for 30 min to label cysteine free-thiols on proteins. Then the plasma was incubated with Laemmli buffer and boiled at 95 °C for 5 min. The proteins were subjected to SDS-PAGE, and BPM-bound proteins were visualized using anti-biotin-HRP-linked antibody (#7075, Cell signaling). For identification of Gpx3, the gel was stained with coomasie brilliant blue (Nacalai), and then dehydrated by 30% acetonitrile, and proteins were reduced by dithiothreitol (100 mM), alkylated by iodoacetamide (100 mM) and digested by Trypsin-Gold (Promega) with protease MAX surfactant (Promega). Peptides were analyzed with liquid chromatography–mass spectrometry (EASY-nLC1000 and Orbitrap Elite, Thermo). Data were analyzed by Mascot ver.2.5.1 (Matrix science).

### Statistical Analysis

Results are presented as the mean ± s.e.m. from at least three independent experiments. Statistical comparisons were made using Student’s *t*-test (for two groups) or analysis of variance followed by Tukey’s post hoc test (for multiple groups). Values of P < 0.05 were considered significant.

## Electronic supplementary material

Supplementary information
