# Supplementary material for: TRPC6 counteracts TRPC3-Nox2 protein complex leading to attenuation of hyperglycemia-induced heart failure in mice
Source: Sci Rep. 2017 Aug 8;7:7511. doi: 10.1038/s41598-017-07903-4 (PMC5548754; doi:10.1038/s41598-017-07903-4)
Supplement: Supplementary file 1 — Supplementary information [file 41598_2017_7903_MOESM1_ESM.pdf]

Supplementary Information for

TRPC6 counteracts TRPC3-Nox2 protein complex leading to attenuation of hyperglycemia-induced heart failure in mice

Sayaka Oda, Takuro Numaga-Tomita, Naoyuki Kitajima, Takashi Toyama,

Eri Harada, Tsukasa Shimauchi, Akiyuki Nishimura, Tatsuya Ishikawa,

Yoshito Kumagai, Lutz Birnbaumer, Motohiro Nishida

a

GPX3_MOUSE Coverage Map

```
1      MARILRASCL  LSLLLAGFVP  PGRGQEKSKT  DCHGGMSGTI  YEYGALTIDG
51     EEYIPFKQYA  GKYILFVNVA  SYUGLTDQYL  ELNALQEELG  PFGLVILGFP
101    SNQFGKQEPG  ENSEILPSLK  YVRPGGGFVP  NFQLFEKGDV  NGEKEQKFYT
151    FLKNSCPPTA  ELLGSPGRLF  WEPMKIHDIR  WNFEKFLVGP  DGIPVMRWYH
201    RTTVSNVKMD  ILSYMRRQAA  LSARGK
```

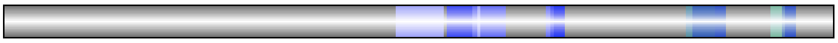

Description	mW (Da)	pI (pH)	Peptides	Theoretical Peptides	Coverage (%)
**Glutathione peroxidase 3 OS Mus musculus GN Gpx3 PE 2 SV 2**	**25257**	8.39	18	14	25.22

b

Precursor MH+ (Da)	Start	End	Sequence	Modifications	Retention Time (min)
1540.78	107	120	(K)QEPGENSEILPSLK(Y)		43.82
1955.02	121	137	(K)YVRPGGGFVPNFQLFEK(G)		56.65
933.49	121	129	(K)YVRPGGGFV(P)		56.65
834.43	121	128	(K)YVRPGGGF(V)		56.61
1022.53	130	137	(V)PNFQLFEK(G)		56.64
818.45	148	153	(K)FYTFLK(N)		47.07
800.44	148	153	(K)FYTFLK(N)		47.05
671.37	149	153	(F)YTFLK(N)		47.03
508.32	150	153	(Y)TFLK(N)		47.05
1300.71	186	197	(K)FLVGPDGIPVMR(W)		53.81
1316.7	186	197	(K)FLVGPDGIPVMR(W)	Oxidation M (11)	47.2
896.5	186	194	(K)FLVGPDGIP(V)		47.11
1040.56	188	197	(L)VGPDGIPVMR(W)		53.83
502.28	194	197	(I)PVMR(W)		53.84
1028.49	209	216	(K)MDILSYMR(R)		53.09
1044.49	209	216	(K)MDILSYMR(R)	Oxidation M (1)	47.4
669.34	212	216	(I)LSYMR(R)		53.04
556.25	213	216	(L)SYMR(R)		53.16

Supplementary Figure 1. Identification of Gpx3 by LC/MS analysis. (**a**) The 25 kDa band was spotted out and subjected to LC/MS analysis. Blue sequence in coverage map indicates identified peptide from the 25 kDa band in comparison with mouse Gpx3 sequence. (**b**) Results of the peptides obtained from 25 kDa bands.

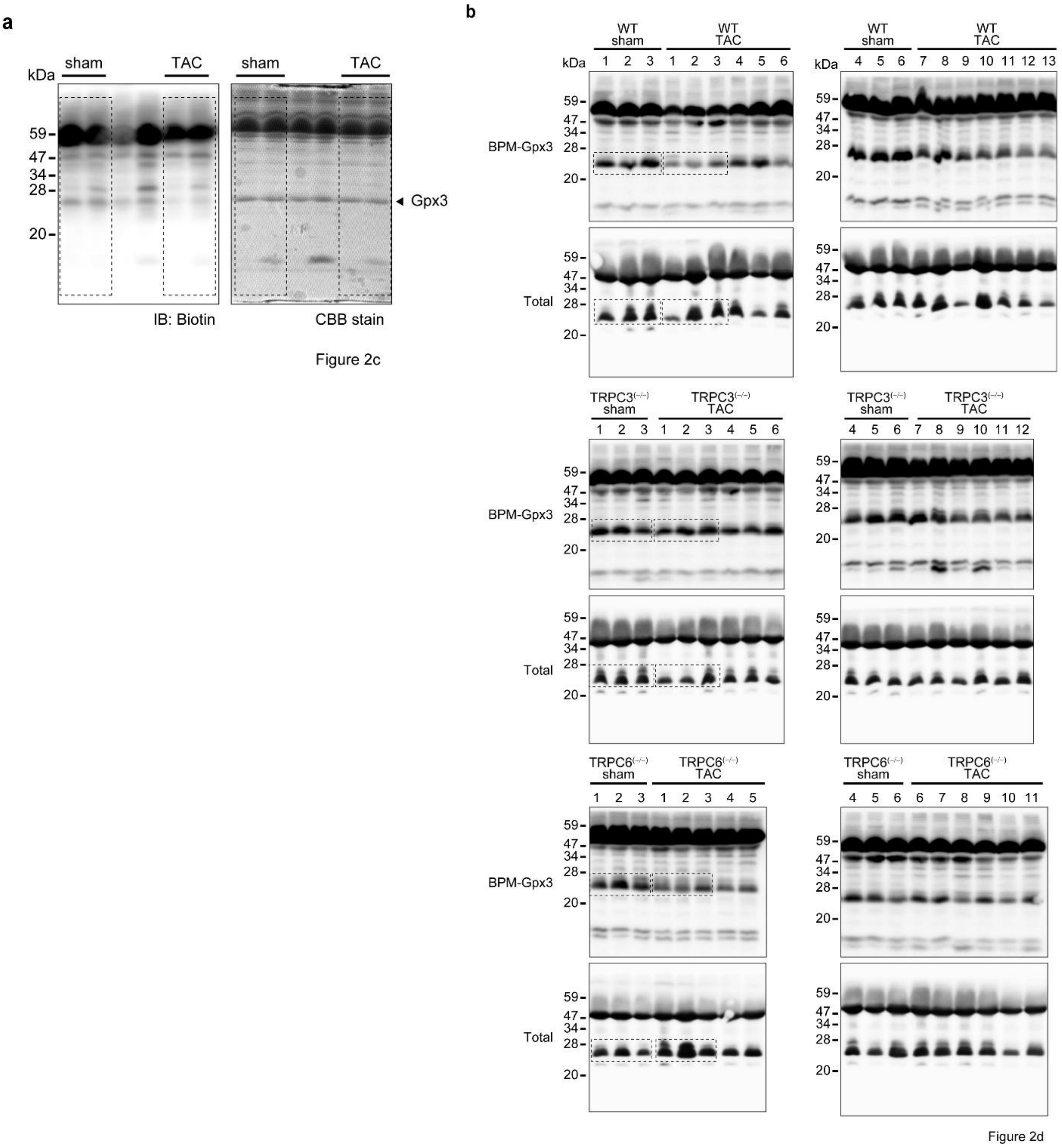

Supplementary Figure 2. (**a**) Full-length blots and gels in Fig 2c. (**b**) Full-length blots of all samples in Fig 2d.

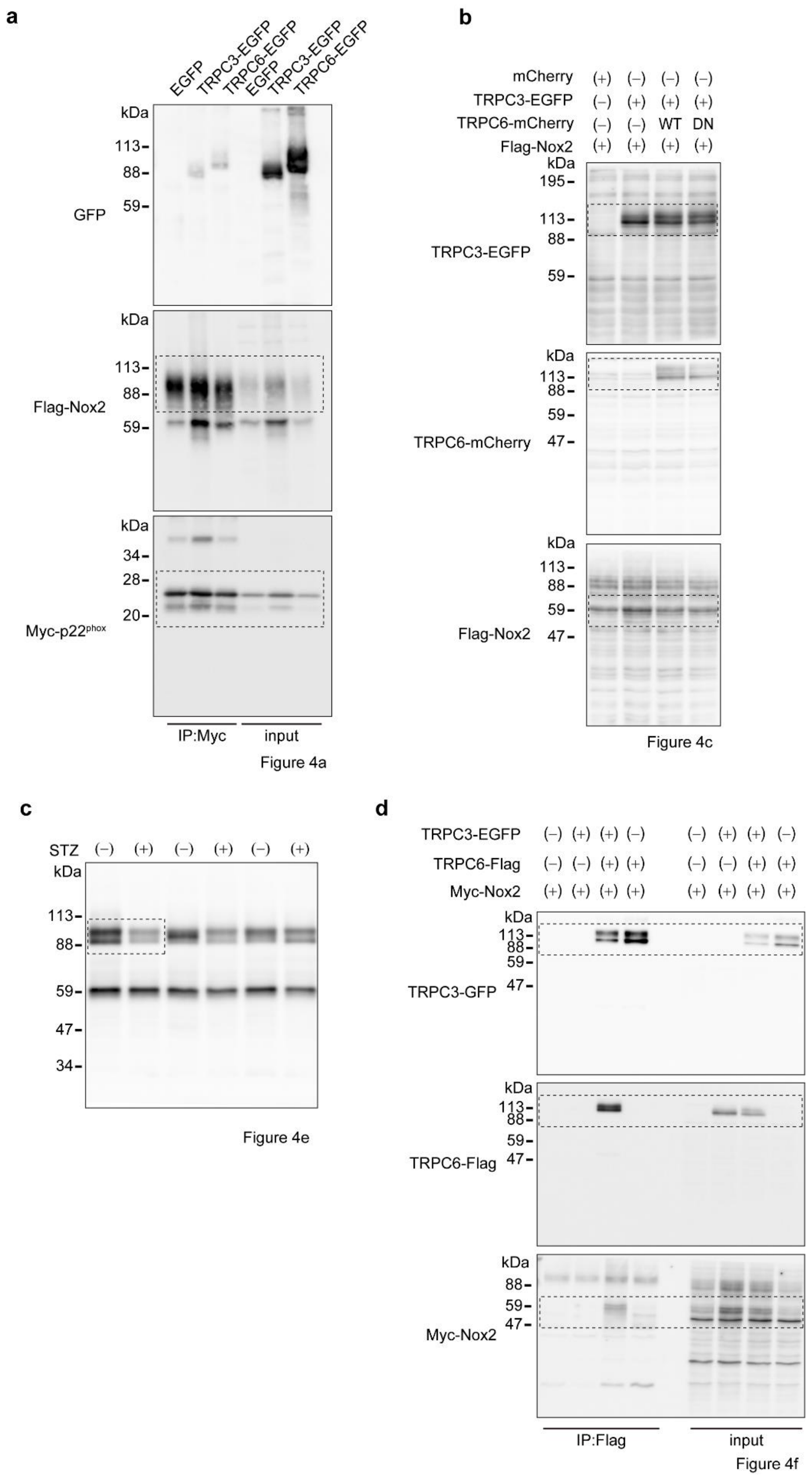

Supplementary Figure 3. (**a**) Full-length blots in Fig 4a. (**b**) Full-length blots in Fig 4c. (**c**) Full-length blots in Fig 4e. (**d**) Full-length blots in Fig 4f.

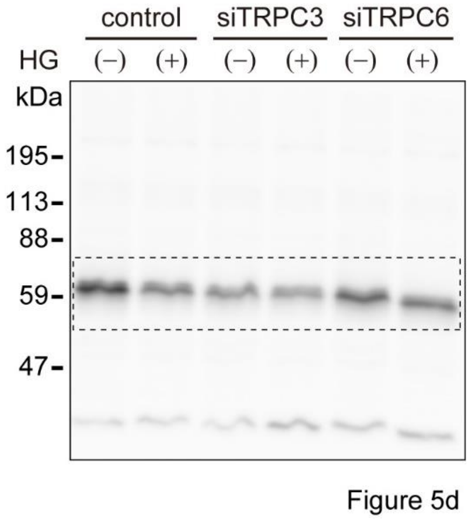

Supplementary Figure 4. Full-length blots in Fig 5d.
